# The effect of artificial gravity on the outcome of a two‐week resistance vibration exercise programme: BRAVE study

**DOI:** 10.1113/EP093066

**Published:** 2025-12-10

**Authors:** Igor B. Mekjavic, Riccardo G. Sorrentino, Jack Fortune, Jason T. Fisher, Lydia Tsoutsoubi, Leonidas G. Ioannou, Andrej Vovk, Matej Supej, Adam C. McDonnell, Urša Ciuha

**Affiliations:** ^1^ Department of Automatics, Biocybernetics, and Robotics Jožef Stefan Institute Ljubljana Slovenia; ^2^ Jožef Stefan International Postgraduate School Ljubljana Slovenia; ^3^ Department of Clinical Physiology Faculty of Medicine University of Ljubljana Ljubljana Slovenia; ^4^ Faculty of Sport University of Ljubljana Ljubljana Slovenia

**Keywords:** exercise physiology, gravity, muscle strength

## Abstract

We evaluated the feasibility and efficacy of a 2‐week training programme comprising resistance vibration exercise (RVE) without and with artificial gravity (AG). Participants (*n* = 24) were divided into three groups: (i) URVE: upright loaded squat exercise; (ii) HRVE: horizontal loaded squat exercise; and (iii) AGRVE: loaded squat exercise conducted on a short‐arm human centrifuge. All participants followed the same protocol and were exposed to the same ground reaction force, whilst performing exercise comprising bilateral squats, triple extension squats and single/bilateral calf raises. Before and after the 2‐week training period, we measured thigh and calf muscle strength with isokinetic dynamometry, muscular power with a jump test, volume with functional muscle magnetic resonance imaging, and body composition with dual‐energy X‐ray absorptiometry. All groups showed significant improvements in eight‐repetition maximum squat strength (*P* < 0.0001, *G* > 0.80), whilst only the AGRVE group demonstrated a small effect in jump height (*G* = 0.26). The AGRVE group significantly increased knee extension and flexion maximum voluntary contraction (MVC), with no comparable changes in the HRVE or URVE groups. The AGRVE group increased total thigh muscle volume (*P* = 0.03), with notable hypertrophy in the vastus medialis, semitendinosus, and vastus intermedius muscles. These findings demonstrate that AGRVE is significantly superior to HRVE and URVE in enhancing knee MVC and thigh muscle volume, thus indicating that artificial gravity improves the outcome of resistance vibration exercise in ambulatory participants.

## Abstract (highlights)

**What is the central question of this study?**
  What is the effect of a 2‐week resistance vibration exercise without and with artificial gravity on muscle mass and function?
**What is the main finding and its importance?**
  Artificial gravity with resistance vibration exercise improved muscle strength and enhanced muscle volume, when compared to the results of similar exercise performed without artificial gravity.

## 1 INTRODUCTION

Exposed to extreme environments, humans and other mammals exhibit phenotypic (during the life of the organism) adaptation to that environment (Amirova et al., 2021; Blaber et al., 2010; Fitts et al., 2011; Goswami et al., 2021; Tanaka et al., 2017). Adaptations in physiological systems attempt to mitigate the deleterious effects of the extreme environment, concomitantly improving performance. The most well‐known adaptations to extreme environments include the improvement of the oxygen‐carrying capacity of blood at altitude and improved heat loss in hot environments (Périard et al., 2015; West, 2004). Similarly, withdrawal of the head‐to‐foot gravitational vector (G_z_) induces adaptations in all physiological systems, but these are generally detrimental (i.e., loss of skeletal muscle mass, bone demineralisation, haemodynamic changes, etc.) (Blaber et al., 2010; Carriot et al., 2021; Grimm et al., 2016; Nagaraja & Risin, 2013; Graham et al., 2021; Shackelford et al., 2004). On Earth, this is observed during prolonged inactivity and unloading of the weight‐bearing limbs (i.e., injury, ageing‐induced inactivity, hospitalisation, etc.). In space, exposure to weightlessness also triggers similar adaptations of physiological systems as inactivity/unloading on Earth (Fernandez‐Gonzalo et al., 2024).

With the advent of longer‐duration space missions on the International Space Station, and particularly in preparation for future missions to Mars, two avenues of research were initiated. One investigates the processes of adaptation to microgravity in different physiological systems, and the other develops strategies to counteract these adaptations (so‐called countermeasures) (Clément, 2011, 2017; Clément, et al., 2015, 2016; Gast et al. 2012). Ground‐based studies have confirmed that a comprehensive and integrative approach is of paramount importance to maintain astronauts’ physical health during long‐duration spaceflight (Fernandez‐Gonzalo et al., 2024; Hajj‐Boutros et al., 2024; Loehr et al., 2015; Rittweger et al., 2005).

To counteract the effects of immobilization, vibration during resistance exercise (RVE) was investigated as a potential countermeasure. Two bed rest campaigns were conducted to evaluate this approach, and the results demonstrated that RVE partially attenuates the physiological deconditioning associated with prolonged immobilisation (Belavý et al., 2010; Mulder et al., 2008, 2015; Rittweger et al., 2006, 2010); however, during the second campaign, it was observed that this is not physiologically different from resistance exercise alone (Belavý, Armbrecht et al., 2010; Belavý, Beller et al., 2011). Nonetheless, vibration proved effective in promoting bone mineralisation.

Another potential countermeasure explored was the use of artificial gravity generated by a short‐arm human centrifuge (SAHC) (Clément, Palowski et al., 2016). This concept is not novel (Noordung, 1929); indeed, numerous studies have documented the implementation of artificial gravity in bed rest investigations (Clément & Traon, 2004; Clément et al., 2015, 2022; Rittweger et al., 2015; Kramer, Kümmel et al., 2020). This intervention was effective in mitigating strength losses during plantar flexion and preserving jumping power; however, it showed no significant effects on other muscular strength parameters or cardiovascular function (Kramer et al., 2020, 2021). As suggested by these results, centrifugation should be combined with strenuous exercise in order to optimize the outcome. As such, a new application is combining resistance exercise, whole‐body vibration and a SAHC. As discussed in previous investigations (Clément, 2011; Frett et al., 2024; Sorrentino et al., 2024, 2025), the multiple force vectors encountered during centrifugation may increase the physical demands of any activity performed under these conditions, as movements must be executed against a complex and multidirectional load acting on the body whilst moving. This multi‐vector resistance requires greater neuromuscular control and higher stabilisation demands, especially during compound and dynamic exercises such as squats (Piotrowski et al., 2018; Sorrentino et al., 2024) or rowing ergometry (Frett et al., 2024). Moreover, due to the short radius of the SAHC (around 2.5 m), even small limb movements can cause shifts in loading due to the distance from the axis.

For this reason, the present study is a prelude to a bigger study (BRAVE: Bed Rest, Artificial gravity and resistance Vibration Exercise) supported by the European Space Agency (ESA), in which the efficacy of resistance vibration exercise (RVE) will be evaluated in the presence of artificial gravity (AG) established with a SAHC, and without it.

The main objective of the present study was to assess the efficacy and feasibility of a 2‐week daily training programme comprising resistance vibration exercise (RVE) in conjunction with artificial gravity (AGRVE) compared to RVE performed upright (URVE) and in a horizontal device (HRVE) in ambulatory subjects. This aspect is particularly relevant considering that astronauts engage in daily physical training; therefore, any proposed countermeasure should closely replicate the training regimen that would be implemented in spaceflight conditions. Assessing the feasibility of a daily exercise protocol is essential to evaluate its tolerability, the potential for excessive fatigue, and any associated risk of injury. Finally, we tested the hypothesis that artificial gravity improves the outcome of RVE in terms of muscle structure and performance, when compared to the results of HRVE and URVE.

## 2 METHODS

This study investigated upright resistance vibration exercise (URVE), in the supine/horizontal position (HRVE), and with artificial gravity (AGRVE). To compare the outcome of the 2‐week training programmes, baseline standard tests regarding muscle structure and performance were performed prior to and after the training.

### 2.1 Protocol

The study was approved by the University of Ljubljana, Faculty of Sports’ Committee for Ethical issues in the field of sport (Reference number: 033‐10/2023‐2). All participants provided their written informed consent to participate in the study, which was performed according to the guidelines of the *Declaration of Helsinki*, excluding clause 35 (i.e., the study was not registered in a publicly accessible database).

**FIGURE 1:**
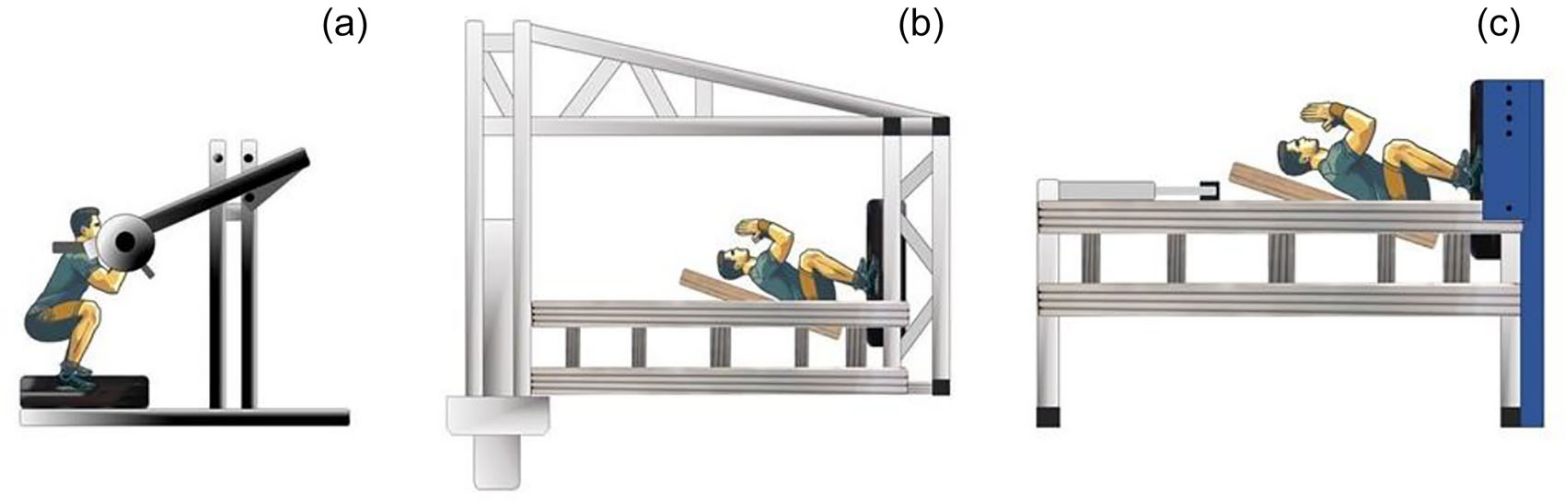
Graphical representation of the devices employed in this study. (a) URVE: upright resistance vibration exercise. Participants performed the exercise with a pendulum‐loaded device whilst standing on a Galileo vibration platform. (b) AGRVE: artificial gravity (AG) and resistance vibration exercise. Participants performed this exercise on a short arm human centrifuge (SAHC). (c) HRVE: horizontal resistance vibration exercise. Participants performed the exercise in a similar manner as the AGRVE group, with the exception that they had to resist a force applied by pneumatic cylinders attached to the sled. The action of the pneumatic cylinders was regulated so that the ground reaction force (GRF) was similar to that on the AGRVE.

Healthy male individuals (*n* = 24) participated in the study. They were equally divided (*n* = 8 in each group) and randomly allocated to three exercise groups (see Figure 1):

- Upright resistance vibration exercise (URVE) group. Participants in this group performed upright loaded squat exercise whilst standing on a rotational vibration platform, as shown in the right panel of Figure 1.
- Horizontal resistance vibration exercise (HRVE) was performed on a bespoke exercise device. Participants in this group performed a loaded squat exercise in a horizontal position, as shown in the left panel of Figure 1. The bespoke exercise device had a sliding platform on which the participant was positioned. This platform had a pivotal mechanism at the level of the hips that allowed the squat exercise to be performed kinematically in the same manner as URVE.
- Artificial gravity and vibration resistance exercise (AGRVE) group. Participants in this group performed the same exercise as the HRVE group, with the exception that the exercise was performed on a SAHC, as shown in the middle panel of Figure 1. The head‐to‐foot gravitational load on the SAHC is dependent on the angular velocity. At any given ground reaction force (GRF), the gravitational load along the axis of the body is not equal as in URVE, but increases linearly from the centre of rotation along the length of the nacelle towards its outer border. Thus, during a squat manoeuvre performed on the SAHC, the gravitational load on the participant will increase during the downwards phase of the squat manoeuvre and decrease during the upwards phase of the squat manoeuvre. This variable head‐to‐foot gravitational load during AGRVE was simulated on the HRVE device by appropriately controlling the force provided via pneumatic pistons attached to the sled mechanism.

During the exercise performed by AGRVE and HRVE, the ground reaction force replicated that observed during URVE.

### 2.2 Participants

The ESA inclusion/exclusion criteria were used in the recruitment of participants (de Villemeur et al., 2023). Participation in the study was subject to the physician's approval. These criteria were chosen because, as mentioned, this study is a prelude for a bed rest (BRAVE study), which will investigate such a population by implementing these criteria.

Following baseline measurements, participants in all three groups attended a 2‐week training session. The weekly training session comprised five daily training sessions, one competition session and a rest day. The intensity of the training session was determined for each participant on the basis of their performance in conducting a loaded squat manoeuvre, whereby the load assigned was determined relative to a calculated one‐repetition maximum loaded squat based on an eight‐repetition maximum test (individual training loads are presented in Table 1). With the exception of the competition day, the daily exercise was kept constant during all training sessions for a given week. During training, all participants were asked to wear gym clothing. Participants’ physical characteristics are presented in Table 2.

**TABLE 1:** Participants’ individualised load for light (25%) and moderate (40%) sessions expressed as the ground reaction force (GRF, kg) measured on the vibration/force plate.

	**GRF (kg)**							
URVE								
Participant no.	1	2	3	4	5	6	7	8
25%	28	25	22	31	25	28	22	25
40%	45	40	35	50	40	45	35	40
AGRVE								
Participant no.	9	10	11	12	13	14	15	16
25%	31	30	20	19	22	28	20	36
40%	50	48	33	30	35	45	33	58
HRVE								
Participant no.	17	18	19	20	21	22	23	24
25%	27	30	36	24	23	27	32	52
40%	44	49	58	39	36	44	51	83

**TABLE 2:** Participants' mean ± standard deviation age, body mass and height for each group.

**Anthropometrics**	**URVE**	**HRVE**	**AGRVE**
Age (years)	22 ± 1	28 ± 5	28 ± 5
Mass (kg)	76.4 ± 7.5	82.6 ± 12.14	78 ± 7.8
Height (cm)	179 ± 2	179.5 ± 8.22	183 ± 3.8

### 2.3 Training paradigm

The exercise during the training programme was prescribed on the basis of a criterion test performed prior to the onset of the training programme. This criterion test was repeated at the end of the first week to determine the training progression for the second week. Participants’ performance during the criterion test determined the exercise intensity that would be assigned for each participant during the following week. For each group, the personalised exercise prescription was derived in a similar manner.

#### 2.3.1 Criterion test: eight‐repetition maximum squat

The evaluation utilised a barbell progressively loaded with weight plates. Prior to the assessment, each participant underwent a warm‐up, consisting of two sets of bodyweight squats and two sets of barbells only. Initial sets with the barbell were performed at a load equivalent to half the participant's individual bodyweight. Progression was adjusted according to individual preferences. Each set comprised eight repetitions with 3 min of rest in‐between sets. Load increments continued until a participant was unable to perform the squat correctly (i.e., achieving a minimum of 90 degrees of knee flexion between the thigh and lower leg). If the participant successfully completed the final set with the designated load, it was designated as their eight‐repetition maximum (8‐RM). However, if the participant did not successfully complete the set, the reference load was reduced by 5%. This reference load was used both for exercise prescription for all groups and as a reference to evaluate the effect of the 2‐week training program.

#### 2.3.2 Exercise prescription

The 14‐day training programme comprised (i) normal training days during which the exercise was one of URVE, HRVE or AGRVE, (ii) rest days, and (iii) a competition day. The latter allowed the determination of exercise intensity progression for each participant. The training days comprised exercise of moderate or light intensity, as shown in Table 3. The aim of the former was to provide a higher training stimulus, but still manageable during centrifugation, and of the latter, an active recovery. The aim of the competition day was to assess the participants' capacity to exercise and to adjust the intensity of the moderate and light exercise for the following week of exercise. The exercise protocol was identical for all groups and comprised three key movements: triple extension squats, back squats and calf raises, with the latter performed in alternating foot positions (toes rotated inward and outward). Triple extension squats and back squats were executed with a controlled rhythm of 3 s for both the eccentric (descent) and concentric (ascent) phases, whereas calf raises incorporated a 1 s isometric hold at maximal extension. By the end of the first week, participants were instructed to perform two additional repetitions per set at both intensity levels, provided they could do so without compromising form or safety. In addition to resistance exercise, both groups were exposed to continuous whole‐body vibration (WBV) delivered via a platform positioned beneath their feet. The vibration frequency and amplitude were set at 20 Hz and 3.5 mm, respectively, based on a literature review identifying parameters that balanced participant tolerance with sufficient neuromuscular stimulation (Rittweger, 2010, 2020). Vibrations were activated exclusively during the exercise sets and deactivated during rest periods.

**TABLE 3:** Weekly training schedule.

Moderate intensity	Aim: optimise hypertrophic responses
Light intensity	Aim: active recovery‐orientated session
Competition day	Aim: assess participant capacity to exercise form and tolerability and adjust intensity
Rest day	Aim: passive recovery day

	Day 1	Day 2	Day 3	Day 4	Day 5	Day 6	Day 7
	Rest	Moderate	Moderate	Light	Moderate	Light	Competition
Week 1	Rest	CM	CM	CM	CM	CM	Competition
Week 2	Rest	CM	CM	CM	CM	CM	Competition

CM = Countermeasure (the exercise performed i.e., URVE (upright resistive vibration exercise), HRVE (horizontal resistive vibration exercise), and AGRVE (artificial‐gravity resistive vibration exercise).

The exercise regimen for any given day is presented in Table 4. Following a warm‐up, the URVE, HRVE and AGRVE exercises comprised a pre‐determined number of repetitions (reps) and total number of sets. Each light and moderate exercise session comprised triple extension squats, bilateral squats and calf raises. The load was set by adjusting the ground force reaction. On the sledge system (HRVE) this was achieved with pneumatic pistons that exerted a force pushing the participant onto the vibration/force platform, and on the centrifuge (SAHC) this was achieved by individually adjusting the angular velocity to obtain the required load assigned for each participant.

**TABLE 4:** RVE and RVE+AG exercises programme detailed description.

	Reps	Sets	Load	Recovery sets	Recovery exercises
Light programme					
Triple extension squat	10	4	25% of 1‐RM	60 s	120 s
Bilateral squat	10	4	25% of 1‐RM	60 s	120 s
Calf raises	10	4	1.3 × BW	60 s	120 s
Moderate programme					
Triple extension squat	8	4	40% of 1‐RM	60 s	120 s
Bilateral squat	8	4	40% of 1‐RM	60 s	120 s
Calf raises	8	4	1.5 × BW	60 s	120 s

BW: = body weight; RM = repetition maximum.

For the URVE, participants performed exercise using a pendulum device, with weights positioned on their shoulders, as shown in the left panel of Figure 1. Load adjustments were made by adding or removing weight plates from the sides of the device. In order to simulate the constant ground reaction force (GRF) experienced by participants in the AGRVE group, individuals in the URVE group maintained the weight placed on their shoulders during the rest breaks between sets.

The HRVE was performed on a bespoke exercise device (right panel in Figure 1), replicating the mechanism on the SAHC (middle panel of Figure 1). Namely, the device comprised a moveable sled system on which the participant lay in the supine position. The sled rotated at hip level, allowing the simulation of the squat manoeuvre as performed in the upright squat exercise (URVE). Pneumatic pistons connected to the sled system provided the force that the participants had to overcome during the squat manoeuvre. The force in the head‐to‐foot direction was not constant. The applied force increased during the eccentric (down) phase and decreased during the concentric (up) phase of the squat. This variable force pattern during the squat manoeuvre was achieved by regulating the pressure in the pneumatic cylinders with a software‐controlled valve. The bespoke software regulated the valve so that the pressure within the pneumatic cylinders exerted a force on the participants' shoulders, resulting in similar ground reaction forces and loading characteristics as experienced during artificial gravity with resistance exercise AGRVE. Thus, variable GRF was established by a control‐system‐regulated pressure valve rather than directly modulating ground reaction forces. The comparison was based on GRF data obtained from individualised training loads measured during exercise.

For resistance vibration exercise whilst exposed to artificial gravity (AGRVE) on the SAHC, AGRVE training was performed on the SAHC at the Gravitational Physiology Laboratory maintained by the Jozef Stefan Institute. This is part of the ESA ground‐based facility in Planica (Rateče, Slovenia). Prior to the exercise, participants were secured to the sled on the SAHC nacelle with a harness to mitigate lateral movement during centrifugation. During the exercise, participants were in constant contact with the operators via a communication system. In addition, three cameras mounted on the nacelle provided visual observation of their face, knees and the position of their feet on the vibration platform. During a familiarisation session, the angular velocity of the SAHC was adjusted so that the GRF measured on the vibration platform matched the GRF recorded in the upright position. Specifically, we matched each individual's 25% and 40% one‐repetition maximum (1‐RM) squat, calculated from the 8‐RM results at the deepest part of the squat. However, this was an approximation, given that the acceleration vector generated by the SAHC is not equal throughout the length of the nacelle, and thus it makes it complex to directly compare to an upright counterpart (Clément, 2011; Clément et al., 2015; Sorrentino et al., 2024). The magnitude of the vector increases as the distance from the central pivot increases, as shown in Figure 1 (Sorrentino et al., 2024, 2025).

### 2.4 Equipment

#### 2.4.1 Short‐arm human centrifuge

**FIGURE 2:**
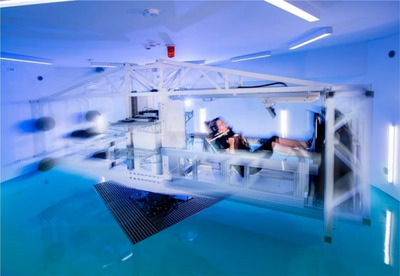
Short arm human centrifuge (SAHC). The participant is lying on the sledge system (same as in the middle panel of Figure 1) with the feet on the vibration/force platform. As seen in the photo, the participant is performing a squat manoeuvre (photo credit: K. Bidovec and A. Hodalic).

RVE was performed on a new generation SAHC (Redwire, Belgium, Kruibeke), shown in Figure 2. The SAHC has two nacelles: one with a sledge system (designed and developed by Amst, Ranshofen, Austria) on which the participant is fastened in the supine position, and another which acts as a counterweight. During centrifugation, participants positioned their feet on a force/vibration platform (Novotec, Germany, Pforzheim). This allowed the GRF to be monitored continuously. The maximum angular velocity of the SAHC is such that it can provide a gravitational force of 4 g on the force/vibration plate. During the study, it was kept to 2 g for safety. The plate can generate vibrations up to 35 Hz, with displacements increasing laterally from the central axis of the plate. The vibration was maintained at 20 Hz during all training sessions. The position of the feet on the plate varied between participants, but in the majority of cases, the feet were about 40 cm apart, which corresponded to 3.5 mm of amplitude. The protocol involved continuous centrifugation without interruptions between sets and exercises, with each session lasting approximately 30 min. Participants consistently performed the training within their assigned time slots, scheduled between 14.00 and 19.30 h.

#### 2.4.2 Horizontal resistance vibration exercise (HRVE) device

The HRVE device was designed and constructed (Mak d.o.o., Spodnje Senice, Slovenia) to replicate the exercise in the SAHC. As explained above, this was achieved by regulating the force delivered by the pneumatic pistons, such that the participant experienced the same ground reaction force as on the SAHC.

#### 2.4.3 Instrumentation

During the exercise training in all three conditions, we monitored participants’ heart rate and electrocardiogram. Participants were instrumented for non‐invasive measurement of arterial pressure (volume clamp method), cardiac output and stroke volume using a Finapres Nova (Finapres Medical Systems, Enschede, Netherlands). A pulse oximeter was positioned on the index finger for the measurement of oxyhaemoglobin saturation. During the exercise, participants were requested to provide a rating of motion sickness (on a scale of 0–4).

### 2.5 Assessment of muscle strength and mass

#### 2.5.1 Isometric maximum voluntary contraction

The maximum voluntary contraction (MVC) was recorded with an isokinetic dynamometer (Biodex System 4 Pro, Shirley, NY, USA) for each of four muscle groups: flexor and extensor muscles of the knee and ankle.

The isometric extension and flexion contractions were performed with the participants in the sitting position for the knee and for the ankle. Both tests were performed on the dominant leg. The participant was firmly strapped to the chair during the isometric MVC test. Prior to the commencement of testing, the system underwent calibration procedures to ensure accurate measurements. The test protocol was similar for each muscle group: after a short warm‐up phase, the participants were requested to perform a maximum extension, followed 30 s after by a maximum flexion, and 30 s after by another pair of extension and flexion contractions until three complete sets of extension/flexion contractions were recorded. Each contraction lasted 5–7 s and there was a 2‐min rest between the successive sets of three contractions. The total duration of the isometric MVC was 15 min per pair of agonist/antagonist muscle groups.

The measured parameters were the maximal isometric torque (units N m) for extension and flexion of the different tested muscle groups. For data analysis, the best result from both contractions was used.

### 2.6 Vertical jump test

Vertical jump performance was measured with a Leonardo Mechanography platform (Novotec Medical GmbH, Pforzheim, Germany). Following a brief warm‐up consisting of squats, the participant was instructed to stand on the platform with hands positioned on the hips and perform a maximal vertical jump. The fixed hand placement was intended to avoid the influence of arm swing and isolate lower‐limb performance. Three maximum effort jumps were performed with 1 min rest between jumps. If the last jump was the highest, the test was continued until a plateau in performance was observed. The plateau was then used for data analysis. The measured parameters are the specific peak power, velocity and the jump height.

### 2.7 Dual energy X‐ray absorptiometry for bone density and body composition

Dual energy X‐ray absorptiometry (DEXA) is a standard clinical technique to assess bone mineral density and body composition. DEXA can distinguish between hard tissue (bone) and soft tissue. Soft tissues can be distinguished further as either lean tissue or fat. In this study, DEXA (Hologic QDR 4500 W, General Electric Healthcare, USA, Chicago, IL) was used to assess participants’ body composition, before and after the 2‐week training period. The total radiation dose was 0.003 mSv per scan. Scans were performed by positioning participants supine on the device with arms resting at the side of the trunk without touching it, and with legs straight and positioned apart, and with toes rotated inward.

### 2.8 Muscle functional magnetic resonance imaging of the lower extremity

Muscle functional magnetic resonance imaging (mfMRI) of the lower extremity was obtained with a 3.0 T MRI with a 12‐channel coil Philips Achieva TX scanner (Philips Healthcare, Best, The Netherlands) MRI scanner. The mfMRI sequences recorded transverse images from the right leg, hip and lower trunk, reaching at least from the foot to the mid lumbar spine. A T1‐weighted DIXON‐sequence (with 6 echoes or 2 echoes, depending on MRI) provided images containing contrast for the identification of different muscles and also information about fat fraction. The imaging protocol consisted of T1 and T2‐weighted turbo spin echo sequences with the following parameters: repetition time (TR) = 2000 ms, echo time (TE) = 13 ms (first echo) with six additional echoes acquired with an echo spacing (ΔTE) of 13 ms (last TE = 78 ms), acquisition matrix = 560×560, flip angle = 90°, voxel size = 0.286×0.286×2.2 mm^3^, number of slices = 14 and SENSE acceleration factor = 2.5. The 14 slices were recorded 10 cm below and above the patella, for, respectively, the calf and the thigh.

### 2.9 Data analysis

Data from each test were acquired using the respective software associated with the instrumentation utilised (such as DEXA, Biodex, mfMRI). Subsequently, the data were exported into Microsoft Excel for organisation and underwent analysis using GraphPad Prism 10 (GraphPad Software, Boston, MA, USA), which also facilitated graph generation. To detect potential outliers, considering the intervariability in participants' responses to training, *Z*‐score analysis was applied to all PRE and POST datasets. Data points with *Z*‐scores exceeding −2 or +2 were identified as outliers and consequently excluded from further analysis. *Z*‐score analysis revealed one outlier in the RVE group for knee extension MVC; thus, it was excluded from the analysis of this specific parameter. Given the relatively low sample size, pre‐ and post‐comparisons were performed using a mixed‐effects model analysis; if a significant effect was found in one of the factors of the model, a further multiple comparison test was performed (Šidák's test). Both for the model and the multiple comparisons test, the significance was set a priori at *P* < 0.05. The effects in the model included two factors: (1) the time of measurement, that is, PRE and POST training regimen (referred to as ‘time’), and (2) the type of training performed (referred to as ‘exercise’). Additionally, to complement the analysis of variance results, an assessment of effect size was undertaken. Due to the relatively small sample size in this study, a Hedge's G test corrected for samples below 20 individuals was utilized (Sullivan & Feinn, 2012).

## 3 RESULTS

All participants completed the 2‐week training programme. All improved their form and tolerability to the exercise programme at the end of the first week of training, so that the exercise intensity was increased during the second week of training.

### 3.1 Motion sickness

Three participants in the AGRVE group experienced motion sickness (three out of four) on the first day of training, and the exercise session had to be terminated prematurely. However, on the fourth day of training, all AGRVE participants were able to complete the entire session. Although participants perceived motion sickness during the first week, this abated towards the end of the week, and no further symptoms were reported during the second week of training. No motion sickness or sopite‐related symptoms were reported during upright or horizontal exercise. No further symptoms occurred after day 3, indicating adaptation. Similarly, initial signs of sopite syndrome, including sweating and light‐headedness, were slightly more pronounced but diminished within the first few days, suggesting successful acclimatisation to the experimental conditions.

### 3.2 Criterion test (8‐RM) and vertical jump height

All groups improved in the 8‐RM squat test. The mixed model analysis revealed a main effect of *time* (*P* < 0.0001), with an average increase of 12%, as well as an effect size analysis (*G* > 0.80).

**FIGURE 3:**
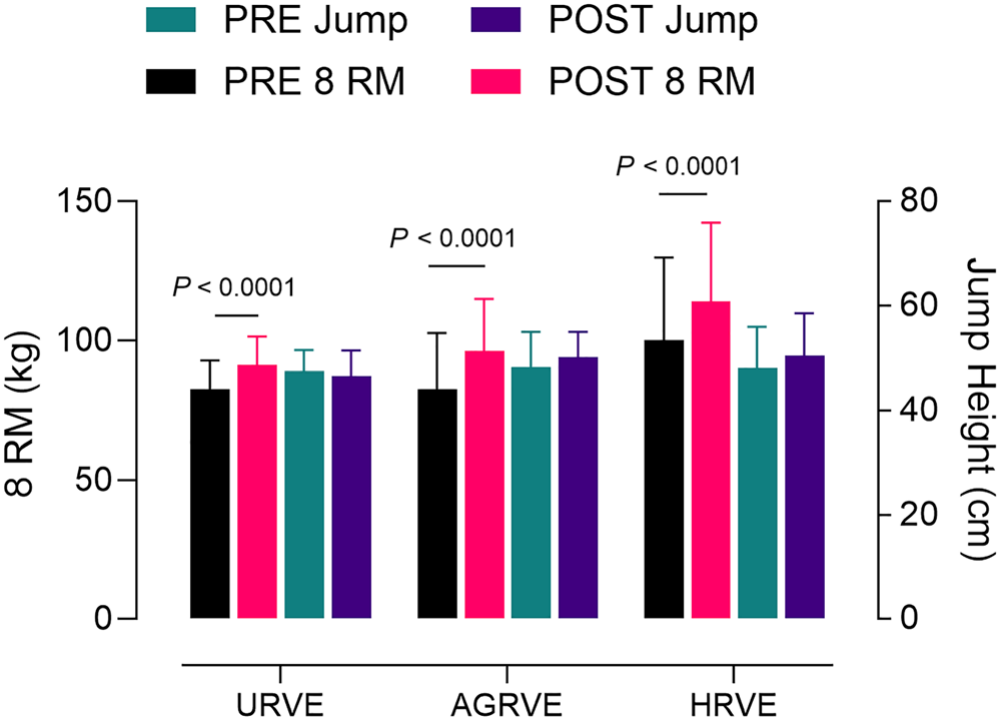
Group comparison bar plots depicting mean ± SD of 8‐RM test (left *y*‐axis) and jump test (right *y*‐axis). *P*‐value refers to the main time effect.

No statistically significant differences were recorded amongst groups for jump height. Effect size analysis revealed a small effect (*G* = 0.26) only for AGRVE. A graphical representation of both squat and jump tests is presented in Figure 3.

### 3.3 Maximum voluntary contraction

#### 3.3.1 Knee strength

Mixed effects model revealed a significant *exercise* × *time* interaction (*P* = 0.0005) on the MVC of knee extension on the sagittal plane. Multiple comparisons test revealed significant differences between PRE and POST of the AGRVE group (230.41 ± 36.63 to 261.66 ± 39.41; *P* = 0.0005) but not for HRVE and URVE.

**FIGURE 4:**
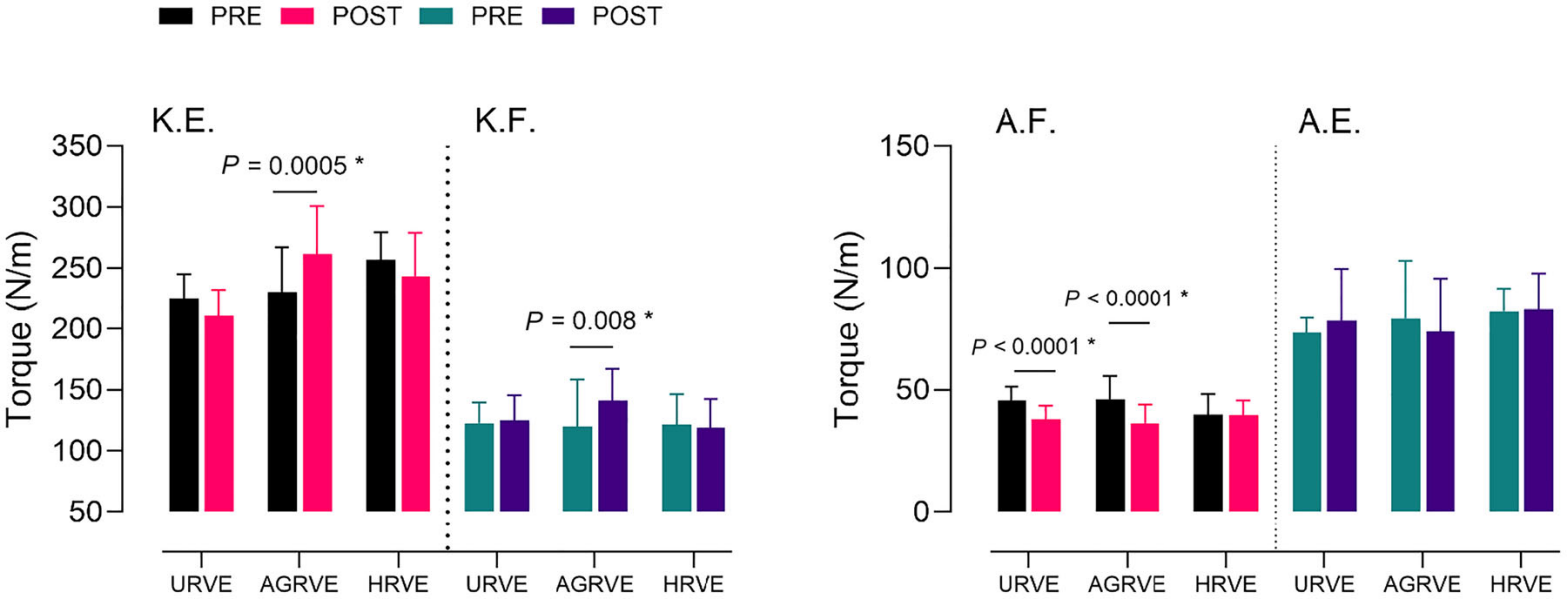
Group comparison bar plots depicting mean ± SD of isometric maximal voluntary contraction for knee extension (K.E.), knee flexion (K.F.), ankle flexion (A.F.) and ankle extension (A.E.). The asterisk (*) represents an exercise × time interaction with *post hoc* analysis value.

Knee flexion MVC analysis revealed a significant *exercise* × *time* interaction (*P* = 0.04). Multiple comparison test revealed a significant difference between PRE and POST of AGRVE group (119.82 ± 38.59 to 141.25 ± 26.02; *P* = 0.008) but not for HRVE and URVE (Figure 4).

#### 3.3.2 Ankle strength

The mixed effects model revealed no discernible differences amongst all the factors (exercise, time) and their interaction.

In contrast, an *exercise* × *time* interaction (*P* = 0.0001) was recorded for ankle flexion MVC. Subsequent analysis revealed a decreased performance for AGRVE (46.27 ± 9.46 to 36.40 ± 7.72) and URVE (45.70 ± 5.80 to 37.96 ± 5.62) groups (*P* < 0.0001), but not for HRVE (Figure 4).

### 3.4 Body composition

No statistically significant differences were recorded for whole‐body lean mass. A main effect of *time* was recorded for lean upper leg mass (*P* = 0.02) and lean lower leg mass (*P* = 0.04).

**FIGURE 5:**
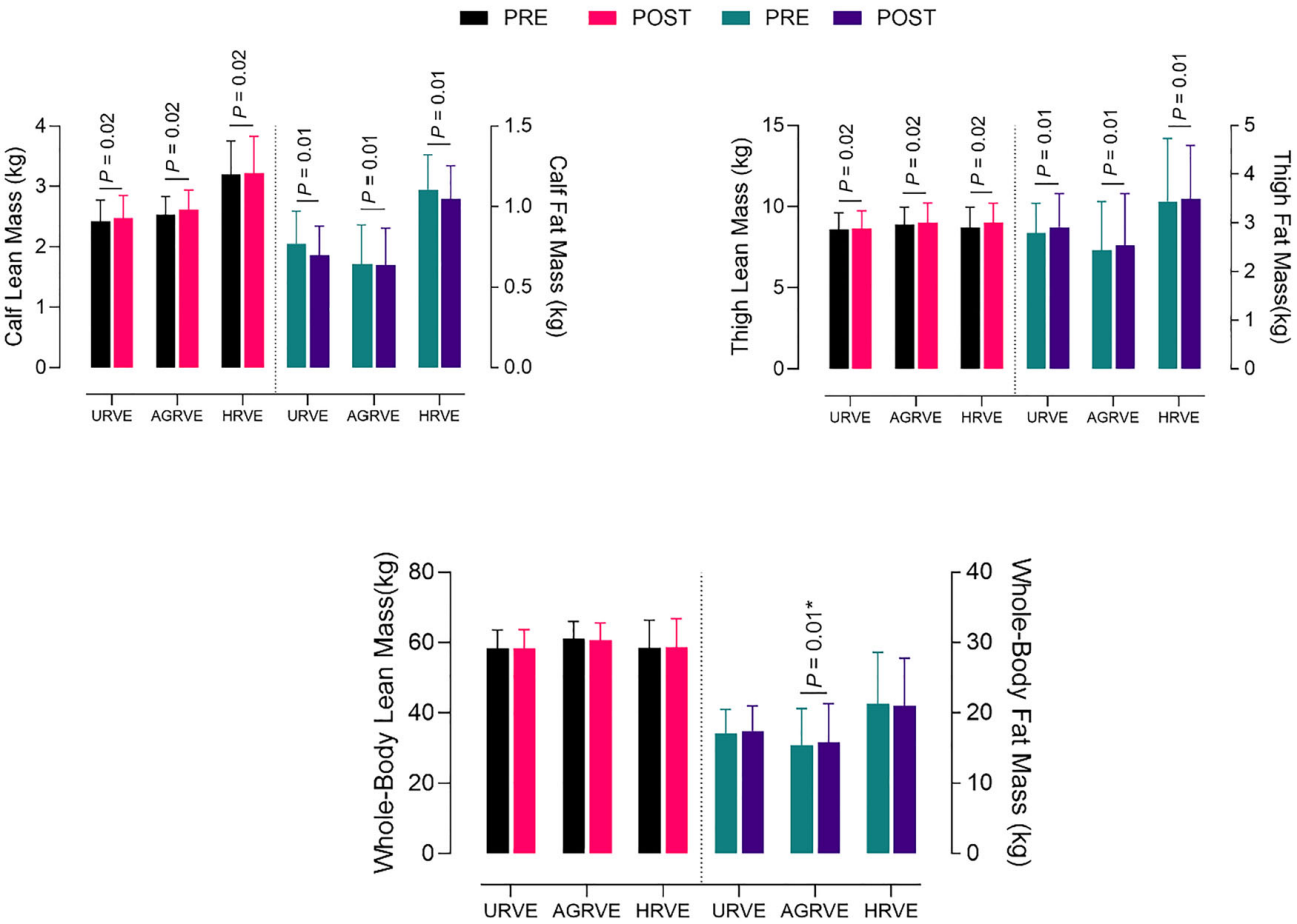
Group comparison bar plots depicting mean ± SD of body composition measured with DEXA scans. *P*‐value refers to the results of the main effect of time. *P*‐value with an asterisk (*) refers to a time × exercise interaction with *post hoc* analysis.

A significant *exercise* × *time* interaction (*P* = 0.01) was recorded for whole‐body fat mass, and subsequent analysis revealed a statistically significant difference between PRE and POST only for AGRVE (15.35 ± 5.27 to 15.86 ± 5.45; *P* = 0.01). A main effect of *time* was recorded for fat upper leg mass (*P* = 0.03) and fat lower leg mass (*P* = 0.01) (Figure 5).

### 3.5 Muscle volume

**FIGURE 6:**
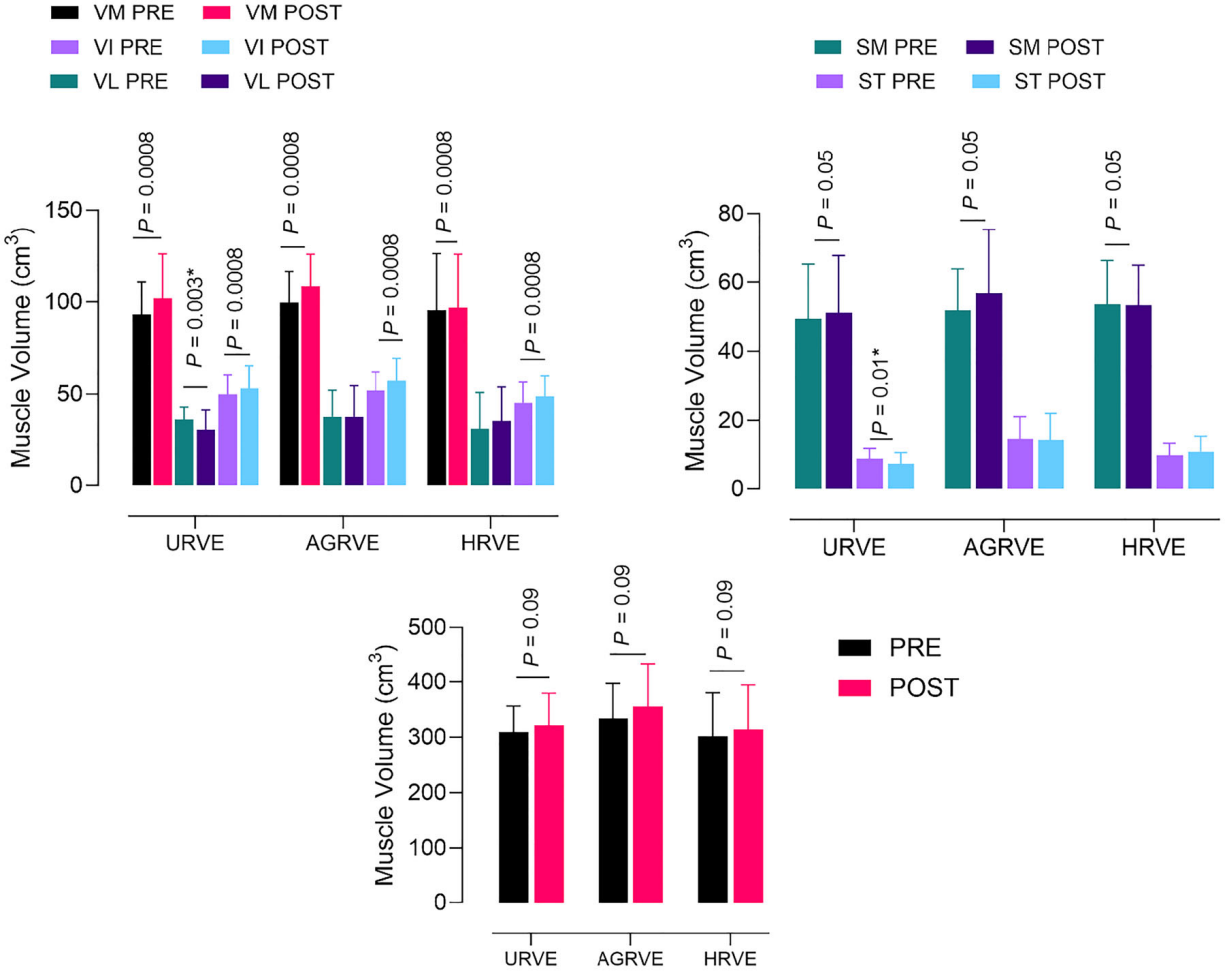
Group comparison bar plots depicting mean ± SD of muscle volume of main anterior thigh muscles (top left panel), main posterior thigh muscles (top right panel) and whole‐thigh (bottom panel). *P*‐value indicates a main effect of time. An asterisk (*) indicates an exercise × time interaction with *post hoc* analysis value. Statistically significant differences between PRE and POST within group. SM, semimembranosus; ST, semitendinosus; VI, vastus intermedius; VL, vastus lateralis; VM, vastus medialis.

A main effect of *time* was found for the vastus medialis (*P* = 0.0008), semitendinosus (*P* = 0.05) and vastus intermedius (*P* = 0.0008). An *exercise* × *time* interaction was found for vastus lateralis (*P* = 0.02) and semitendinosus (*P* = 0.01). Subsequent analysis revealed a decrease in volume in vastus lateralis (36.12 ± 6.88 to 30.75 ± 10.77) and semitendinosus (8.79 ± 3.19 to 7.32 ± 3.23), but only in URVE (respectively, *P* = 0.01 and *P* = 0.03). A main effect of *time* was found for semimembranosus (*P* = 0.05). Total thigh volume was calculated by summing all individual muscle volumes. A main time effect was found for total thigh volume (*P* = 0.009) (Figure 6).

**FIGURE 7:**
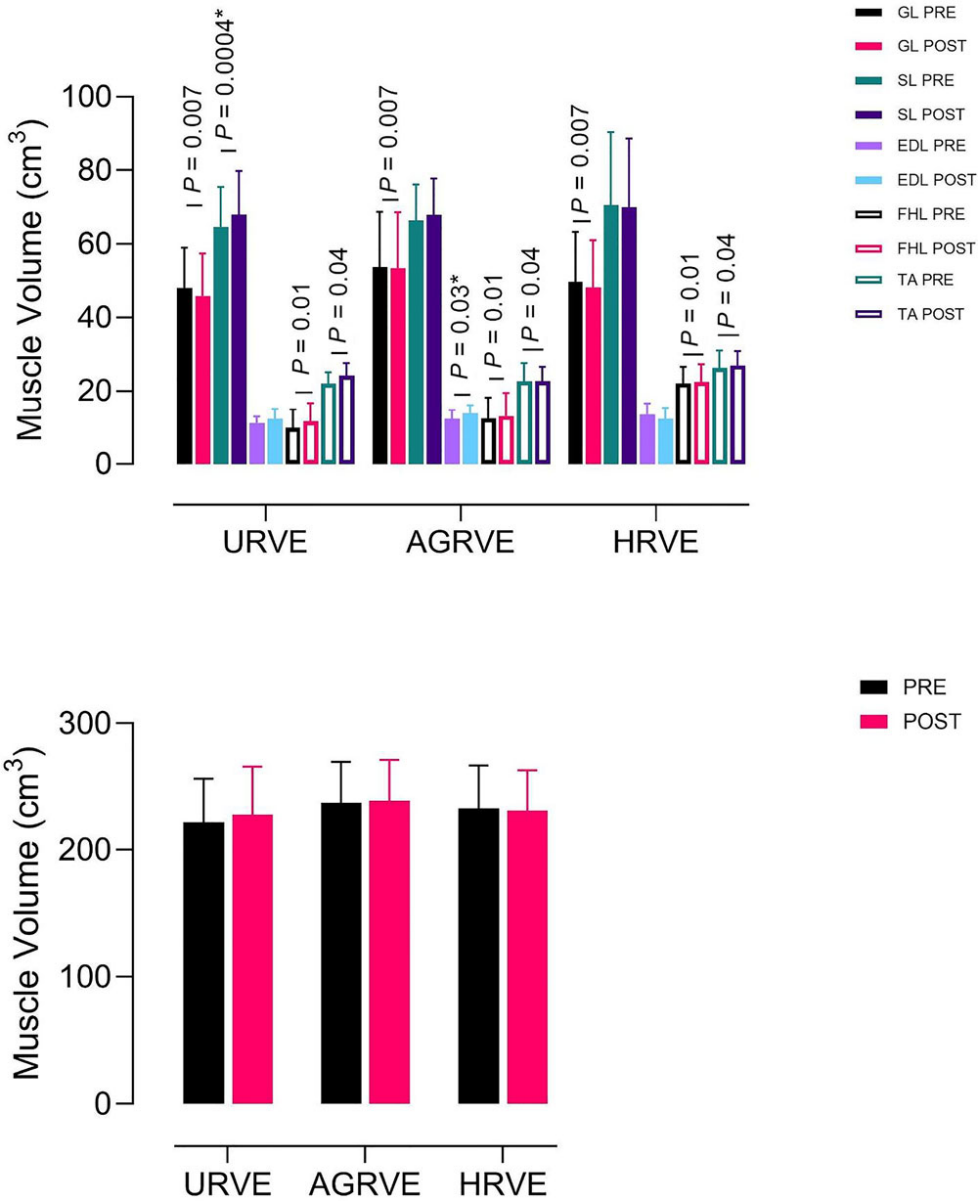
Group comparison bar plots depicting mean ± SD of calf muscle volume (top panel) and whole calf (bottom panel). An asterisk (*) indicates a statistically significant difference between PRE and POST within group. EDL, extensor digitorum longus; FHL, flexor hallucis longus; GL, gastrocnemius lateralis; SL, soleus; TA, tibialis anterior.

A main effect of *time* was found for gastrocnemius lateralis (*P* = 0.0007), flexor hallucis longus (*P* = 0.01) and tibialis anterior (*P* = 0.04). An *exercise* × *time* interaction was found for soleus (*P* = 0.008) and extensor digitorum longus (*P* = 0.01). Subsequent analysis revealed an increase in volume for soleus in URVE (64.68 ± 10.83 to 68.02 ± 11.87; *P* = 0.0004) and an increase in volume for extensor digitorum longus in AGRVE (12.37 ± 2.38 to 13.85 ± 2.28; *P* = 0.03), as presented in Figure 7.

## 4 DISCUSSION

The aim of the present study was to investigate the feasibility and efficacy of an AGRVE exercise programme, with a particular emphasis on the possibility of using this countermeasure during space missions. After several sessions, participants were able to tolerate the AG protocol and consistently complete the daily training regimen. Compared to horizontal and upright RVE, AGRVE improved knee maximal voluntary contraction. Muscle volume responses were variable across conditions, whilst no significant differences between exercise modalities were observed for other measured parameters. These outcomes are likely attributable to the unique multi‐vectorial loading environment provided by centrifuge‐based exercise, which is not replicated in either upright or horizontal RVE.

### 4.1 Training

#### 4.1.1 Exercise modality

The primary distinction between centrifugation and upright exercise lies in how the load is distributed and experienced by users. During centrifugation, the load (acceleration) increases along the length of the nacelle and is not constant through the body (Clément, 2011; Clément & Traon, 2004; Sorrentino et al., 2024). For instance, a participant experiencing 1 *g* at the centre of mass whilst supine on a centrifuge would encounter higher loads (∼2 *g*) at the feet and lower loads (∼0.5 *g*) at the head. Furthermore, when centrifugation is combined with dynamic movements, the centre of mass shifts horizontally throughout the range of motion. Specifically, during the squat manoeuvre, the gravitational forces experienced by the participant will vary throughout the downward and upward phases of the squat manoeuvre (Sorrentino et al., 2024). In contrast, during URVE, the load remains constant from the head to the feet. Thus, these differences in load distribution between URVE and AGRVE should be considered when comparing the outcomes of a training programme. The variable load experienced by participants during AGRVE was replicated, to a degree, during HRVE. Specifically, the pneumatic pistons providing the resistance to the squat manoeuvre were programmed to replicate the forces experienced by the participants in the AGRVE. In particular, GRF during the HRVE and AGRVE was similar.

The selected training load for this study was set to be manageable for participants during centrifugation, whilst still providing a training stimulus. Whilst this proved effective for the AGRVE group, it was insufficient for HRVE and URVE. The findings from the URVE and HRVE groups partially align with those observed in previous studies. Jenkins and colleagues (2016) showed that training three times per week at 30% of 1‐RM does not yield neuromuscular improvements over periods of 2 and 4 weeks. However, differences exist between this study and Jenkins' in terms of the targeted muscles and overall intensity, and therefore, the comparison must consider these variations. In addition, the choice of 40% 1‐RM was intentionally kept below traditional strength thresholds and was not performed to failure. This was based on the hypothesis that the combination of artificial gravity and vibration stimuli would sufficiently enhance the neuromuscular load, potentially compensating for the lower resistance intensity. Whilst prior evidence (Weakley et al., 2023) at similar intensities (e.g., 30–40% 1‐RM) has often involved training to failure, the goal of this study was to investigate whether this combined stimulus could yield positive effects without reaching fatigue or causing muscle injury that would impact the training schedule. For HRVE and URVE, such a load was insufficient to generate any meaningful improvement.

### 4.2 Muscle strength

The increased knee maximal isometric torque observed in the AGRVE, but not in the URVE and HRVE groups, may be attributable to the overall acceleration vectors that are generated in AGRVE. Namely, centrifugation generates more acceleration vectors compared to an upright exercise (Clément, 2011; Clément et al., 2015; Sorrentino et al., 2024). During an upright squat, the only gravitational vector acting on the individual is the normal gravity vector (9.80 N). When standing upright, the only gravitational force acting on the human body is that of Earth's gravitational field. Adding an external load produces a proportional increase in the ground reaction force (GRF), yielding a linear relationship with the load magnitude. During upright squatting exercises, the increase in GRF arises from the combined accelerations of body mass and the added load. In the context of AGRVE, where microgravity conditions are simulated by centripetal acceleration, two gravitational components emerge and combine vectorially: one directed radially and one vertically. Their interaction results in a dynamic resultant vector, whose magnitude exceeds that of the individual vectors (Frett et al., 2024, 2014; Kramer et al., 2020; Sorrentino et al., 2024). This vector can be calculated at any reference point, but it is important to note that this value is not constant as it changes dynamically during the exercise (Frett et al., 2024; Sorrentino et al., 2024). Consequently, the total force experienced by an individual with an external load is augmented by both components. Again, squat movements in this environment further modulate the resultant GRF through the accelerations of both the body mass and the external load. It has been previously emphasised (Sorrentino et al., 2024) that the third vector altered the kinematics of squat exercise, and participants had to make an additional effort to complete the movement properly. It was suggested that the additional muscular effort could have resulted in a higher muscular activation and, in the long term, in an enhanced anabolic stimulus compared to conventional exercise. A significant reduction in MVC of ankle plantar flexion was observed in both the AGRVE and URVE groups. Although not statistically significant, a similar trend was also noted in the HRVE group. This decline may be attributed to participants refraining from their habitual physical activity throughout the intervention period. That is, all participants in the study were fairly active and participated in regular activity prior to entering the study. As part of their inclusion in the study, they were requested to refrain from participating in any form of exercise other than the training imposed by us. It is very likely, at least for the majority of the participants, that the daily exercise we introduced did not match their normal activity level. During the 30‐min exposure on the SAHC, the duration of active exercise was only 12 min. The remaining time was the cumulative time for all the rests between sets. During this time, the participants were still exposed to the artificial gravity. The reduced daily activity of the ankle plantar flexors may have caused the observed slight decrease in their maximal force. The training load selected for the calf raise exercise was obviously insufficient to either preserve or promote any increase in muscle strength.

The observed differential effect of activity on the leg muscles is well known. We have previously shown that unloading inactivity, as during bed rest, causes non‐uniform atrophy of the leg muscles, with the muscles of the gluteal region being least affected and ankle plantar‐flexor muscles exhibiting the greatest degree of atrophy. Mulder et al. (2009) also reported that resistance exercise performed during bed rest thrice weekly prevented the loss of thigh muscle mass, but was not capable of preserving calf muscle mass. As emphasised by Mulder et al. (2009), preserving calf muscle mass requires more frequent exercise, and of greater intensity than for the thigh muscles. In the present study, the exercise performed 6 times per week was of insufficient intensity and/or duration to preserve the ankle plantar‐flexion force. Future strength training regimens, particularly focusing on maintaining calf muscle mass and function, should ensure that the resistance training addresses this concern. Nevertheless, the results of the present study demonstrate that AGRVE offers more potential for strength training improvement compared to upright exercise.

Muscular adaptations to any resistance training are dependent on several factors, such as motor unit recruitment synchronisation, number of motor units recruited, antagonist muscle synergetic activation, and more efficient movement technique (Del Balso & Cafarelli, 2007; Del Vecchio et al., 2019). Furthermore, individuals unfamiliar with a particular exercise undergo a process of familiarization with the movement over time, enhancing overall exercise efficiency. After 2 weeks of daily exercise, participants in this study exhibited an improvement in their 8‐RM performance, which is in line with findings of previous studies implementing a squat RM test (Artero et al., 2012). To guarantee that this change was not influenced by external factors, the test was supervised by the same operators and strength and conditioning experts. In contrast, the counter movement jump (CMJ) did not undergo any improvement in both groups, and the effect size recorded for the AGRVE group is not sufficient to claim that there might be a trend towards improvement. In addition, the CMJ test reflects muscle power, and the protocol employed in this study was not designed to specifically improve power.

### 4.3 Muscle volume

Exercise‐induced muscle hypertrophy is a slow process that requires medium to long‐term exercise training programmes (DeFreitas et al., 2011; Roberts et al., 2023). To achieve a given level of hypertrophy, these programmes may vary from weeks to months, depending on nutrition, genetics, training type, and training intensity. There is evidence that supports an increase in muscle cross‐sectional area (CSA) after just two training sessions of exercise performed to failure (DeFreitas et al., 2011), although other studies reported the first noticeable muscle CSA increase after 20 days of training (Seynnes et al., 2007). Narici et al. (1996) reported an almost linear increase for muscles CSA and strength increase over a period of 6 months from the onset of the training stimulus. The current investigation employed an innovative methodology to examine muscle hypertrophy (Sorrentino et al., 2025). MRI analysis utilises multiple image slices to accurately assess muscle volume, a method that has been widely employed in numerous bed rest studies (Belavý, Miokovic et al., 2010; Fuchs et al., 2025; Hides et al., 2007). The novel and previously validated (Sorrentino et al., 2025) approach employed in this study incorporates artificial intelligence tools to calculate muscle volume (cm^3^) from 14 sequential slices of the thigh and calf. This technique offers a volumetric analysis, thereby providing a more comprehensive understanding of muscle morphology.

Our findings concur with previous research, indicating that muscle hypertrophy is not evident as neuromuscular adaptations after only 2 weeks of training; nevertheless, it is still possible to detect volume changes as observed in our results, especially in the AGRVE group.

### 4.4 Neuromuscular adaptation

As discussed previously (DeFreitas et al., 2011), it is commonly supported that initial training adaptations are predominantly neuromuscular, followed by hypertrophic changes. However, the notion that hypertrophic adaptations must await the onset of neural improvements lacks substantial justification, as hypertrophy and neuromuscular adaptations are not mutually exclusive processes, and our results concur with previous research claiming that it is possible to observe some degree of hypertrophy even in the short to middle term of exercise. The improvements over 2 weeks of training are not linear for all muscles. The degree of muscle growth has been observed to differ amongst the individual muscles within a muscle group (Folland & Williams, 2007; Narici et al., 1996; Russell et al., 2000). The variations in hypertrophy across different muscles may be influenced by the degree of individual load and activation experienced during exercise, which can differ amongst participants based on their morphological characteristics and training experience.

### 4.5 Vibration effect

The effect of WBV on muscular strength, power, and activation remains equivocal (Cochrane & Rittweger, 2020; Rittweger, 2010, 2020). Some studies did not find any additional benefit of implementing vibrations and resistance training to improve muscle parameters (Arora et al., 2021; Artero et al., 2012; Roelants et al., 2004), whilst other findings support improved strength and power (Cardinale & Bosco, 2003; Delecluse et al., 2003; Despina et al., 2014). The present study's findings tend to support the hypothesis of no added value of vibration exercise. The difference between these findings and previous research supporting WBV may be due to differences in vibration frequency and the population investigated. Compared to previous studies, this study employed a lower frequency (20 Hz), which remained the same for the entire study; moreover, participants were familiar with physical exercise, and, as has been shown previously, the effect of vibrations on active individuals tends to be non‐significant (Arora et al., 2021). The vibration intensity was selected based on parameters previously reported in the literature to effectively stimulate muscle and bone tissue (Rittweger, 2010). It was adjusted to ensure safety and comfort whilst still being perceived as physically demanding by participants. In addition, preliminary investigations indicated that, during horizontal configurations (AGRVE and HRVE), higher vibration frequencies led to foot slippage on the platform, thereby increasing the risk of injury. The results of this study suggest that the primary factor influencing the observed effects was centrifugation itself, rather than the application of whole‐body vibration alone.

### 4.6 Limitations

The findings of this study should be interpreted in light of several considerations. Notably, early adaptations in muscle structure were observed after only 2 weeks of daily AGRVE training, indicating a rapid physiological response. However, conventional training studies typically involve longer intervention durations, often extending to 6–8 weeks. Therefore, further investigation is warranted to assess the effects of AGRVE over extended training periods. Additionally, participants were instructed to refrain from engaging in any other forms of exercise during the study. However, as they were not confined to a controlled environment, physical activity and nutritional intake were not objectively monitored (e.g., via activity trackers or dietary logs). Consequently, the potential influence of these uncontrolled external variables on the outcomes cannot be ruled out. In conclusion, one limitation of this study is the absence of a true control group without exercise, which restricts the ability to fully isolate training effects from other factors such as time. Additionally, whilst the study compared three distinct exercise modalities, the experimental design was not fully balanced with respect to all combinations of posture and artificial gravity exposure. For instance, a group exposed to artificial gravity without resistance exercise was not included. This may have limited the statistical power to detect subtle interaction effects or differences between conditions. Nevertheless, the study was conceived as a preliminary investigation to inform the design of future, more comprehensive studies.

### 4.7 Conclusions

Based on our findings, AGRVE appears to be more effective than URVE and HRVE in enhancing knee muscle function, with mixed responses for muscle volume and no meaningful effect on body composition. However, these results should be interpreted with caution. The training protocol was designed to be feasible for participants in the centrifuge whilst remaining comparable to the other groups. Due to the unique characteristics of the SAHC, the resistance load was not identical across groups. Future studies should focus on optimising resistance load prescription for SAHC‐based training. Furthermore, different strength and conditioning resistance training techniques should be investigated to optimize SAHC as a training modality.

Despite the many countermeasures developed to prevent adaptation to microgravity (during space travel and sojourns on the International Space Station) and reduced gravity (during sojourns on the Moon and Mars), it is now apparent that intermittent exposure of astronauts to artificial gravity might be a plausible solution for maintaining their health and well‐being, as suggested in 1929 by Herman Noordung.

## AUTHOR CONTRIBUTIONS

Conception and design of the work: Igor B. Mekjavic, Matej Supej, Adam C. McDonnell and Urša Ciuha. *Acquisition of data for work*: Riccardo G. Sorrentino, Jack Fortune, Jason T. Fisher, Lydia Tsoutsoubi, Leonidas G. Ioannou, Andrej Vovk and Urša Ciuha. *Analysis and interpretation of data for work*: Igor B. Mekjavic, Riccardo G. Sorrentino, Jack Fortune, Jason T. Fisher, Lydia Tsoutsoubi, Leonidas G. Ioannou, Andrej Vovk and Matej Supej. *Acquisition of funding*: Igor B. Mekjavic and Urša Ciuha. All authors contributed to drafting and revising the work. All authors have read and approved the final version of this manuscript and agree to be accountable for all aspects of the work in ensuring that questions related to the accuracy or integrity of any part of the work are appropriately investigated and resolved. All persons designated as authors qualify for authorship, and all those who qualify for authorship are listed.

## CONFLICT OF INTEREST

The authors declare no conflict of interests.

## Data Availability

The data are available on request.
